# Postoperative nosocomial COVID-19 infection in surgical patients during pandemic: A prospective observational cohort study

**DOI:** 10.1016/j.amsu.2022.104730

**Published:** 2022-09-30

**Authors:** Narjes Mohammadzadeh, Alireza Abkhoo, Mohammad Ashouri, Amirmohsen Jalaeefar, Amirmasoud Kazemzadeh Houjaghan, Behnam Ghorbani, Sara Ataie-Ashtiani, Mohammadreza Salehi, Ali Jafarian

**Affiliations:** a Department of Surgery, Imam Khomeini Hospital Complex, Tehran University of Medical Sciences, Tehran, Iran; b Department of Surgical Oncology, Cancer Institute, Tehran University of Medical Sciences, Tehran, Iran; c Department of Internal Medicine, Imam Khomeini Hospital Complex, Tehran University of Medical Sciences, Tehran, Iran; d College of Medicine and Public Health, Flinders University, South Australia, Australia; e Infectious Diseases and Tropical Medicines Department, Tehran University of Medical Sciences, Tehran, Iran; f Liver Transplantation Research Center, Imam Khomeini Hospital Complex, Tehran University of Medical Sciences, Tehran, Iran

**Keywords:** Surgery, COVID-19, SARS-Cov-2, elective surgery, Urgent surgery, Pandemic

## Abstract

Background: increased pressure on healthcare systems and possible risk of nosocomial COVID-19 infection during pandemic urged many guidelines to severely restrict the number of operations. The aim of this study was to investigate the risk of COVID-19 infection and its complications in patients undergoing urgent or elective operations.

Methods: a prospective observational cohort study was conducted in a tertiary surgical center and all patients with no preoperative history of COVID-19 undergoing elective or emergent surgeries were included in this investigation. chest computed tomography (CT) scan or polymerase chain reaction (PCR) test were performed on patients before and after surgery.

**Results:**

183 patients who underwent an operation were enrolled in this study. In postoperative follow-up, 12 patients were positive for COVID-19 infection as identified by RT-PCR and non-contrasted chest CT scans. Regrettably, 2 individuals passed with one of these individuals dying as a direct result of COVID-19 infection. All the 12 cases of post-operative COVID-19 patients underwent elective surgeries.

**Conclusion:**

the gathered results indicate a need for the re-evaluation of the risks of operation during the COVID-19 pandemic. If operations are performed while observing protective and preventative protocols, the risk of post-operative nosocomial COVID-19 is significantly reduced. Hence, the consequences imposed on patients by the delay or cancellation of operations (most notably in cancer cases) may outweigh the risk of post-operative COVID-19 infections.

## Highlights

• If operations are performed, considering protocols, the risk of post-operative nosocomial COVID-19 are significantly reduced.
• The risks of delay in operations (most notably in cancer cases) may outweigh the risk of post-operative COVID-19 infections.
• It depends on availability of resources to decide about performing elective surgical procedures during covid-19 outbreaks.

## 1 Introduction

During the December of 2019, an emerging disease conclusively changed health-care systems worldwide. The COVID-19 virus was first identified in the Wuhan region of China and progressed to spread to other nations over the successive months [1]. Accordingly, March of 2020, the World Health Organization announced its recognition of a global pandemic [1,2]. COVID-19 infection presentation ranges from asymptomatic infection to cases of lethal pneumonia [3]. As of March 2021, this virus has regrettably resulted in over 120 million recorded cases of infection and 2.7 million deaths globally [2]. The first case of COVID-19 in Iran was reported on February 19th^,^ 2020 in Qom, and after this, the virus rapidly spread all over the country [4].

Admission of an unprecedentedly high number of patients to unprepared hospitals due to the COVID-19 pandemic resulted in increased pressure on healthcare systems [5,6]. The shortage of hospital and Intensive Care Unit (ICU) beds and established guidelines with the aim of reducing the risk of COVID-19 infections in hospitalized patients significantly reduced the number of operations taking place during the pandemic [7,8]. Furthermore, it was suggested that patients who undergo operations are at a higher risk of nosocomial COVID-19 infection due to increased viral exposure in hospital settings in addition to their immunosuppressed state [9]. Synchronously, literature indicated high mortality and morbidity rate in COVID-19 cases after surgeries [10,11].

The discussed guidelines provided instructions for all aspects of the health care system including health care personnel, patients, transportation processes, operating rooms, and equipment use [12]. Thus, guidelines severely restricted the number of elective operations, and urgent operations were completed non-laparoscopically and with regional anesthesia [[13], [14], [15], [16]]. This was not only due to the high risk of COVID-19 infection but also to the fact that the majority of medical supplies were devoted to COVID-19 management [17]. Similarly, within our referral center, hospital protocols were established and followed, resulting in the cancelation of the majority of elective surgeries [18].

As rapid diagnostic tests for COVID-19 have become widely available and the COVID-19 pandemic has continued, several surgical centers are applying screening systems for COVID-19 testing of patients before admission [19]. If a positive result is obtained, surgeries are postponed or are only performed with special precautions [20]. Despite the discussed guidelines and protocols, the exact risk of COVID-19 infection in admitted surgical cases and the resulting complications has not yet been determined. Consequently, this study aims to investigate the risk of COVID-19 infection and its complications in patients undergoing urgent or elective operations in our tertiary center and assess the safety of performing operations in terms of COVID-19 infection during the pandemic.

## 2 Methods

The main goal of this study is to evaluate the rate of postoperative COVID-19 infection and its resultant complications and mortality to provide a basis for surgical risk assessment, ensuring the delivery of the best medical care to patients a prospective observational study was conducted in a tertiary surgical center. Patients who underwent surgery from April to September 2020 was enrolled in this study. This period coincided with the peak of the COVID-19 pandemic in Iran when many non-urgent operations were canceled. This research is fully compliant with the STROCSS 2021 criteria [21] and registered in the Research Ethics Committees of Vice-Chancellor in Research Affairs of Tehran University of Medical Sciences. The approval ID is IR. TUMS.VCR.REC.1399.216. https://ethics.research.ac.ir/ProposalCertificateEn.php?id=128698&Print=true&NoPrintHeader=true&NoPrintFooter=true&NoPrintPageBorder=true&LetterPrint=true.

All patients over 18 years of age, while they had no evidence of COVID-19 infection undergoing elective or emergent surgeries in a tertiary hospital were included in this investigation. Patients suspected of COVID-19 infection based on preoperative chest computed tomography (CT) scan or positive polymerase chain reaction (PCR) test were excluded from the study. Moreover, patients with a positive COVID-19 test within 3 days after surgery were excluded due to the likelihood of the acquirement of this infection pre-operation. For enrolled patients, suspicious postoperative symptoms followed by a definitive diagnosis based on PCR testing and Chest CT scans 3–14 days post-surgery was considered as positive post-operative COVID-19 infections.

Demographic information and comorbidities were recorded and tabulated. Based on surgical protocols, all patients underwent a non-contrasted chest CT scan but a reverse transcription polymerase chain reaction (RT-PCR) of the oropharynx and nasopharynx was only done for symptomatic patients. Information regarding the operation, including operation type, anesthesia, and operation duration were recorded. All included patients were followed up after surgery and were assessed every day during their hospitalization in addition to follow up appointments 2 and 6 weeks after discharge in person or by phone. During these appointments, they were asked about and examined for suspicious symptoms indicative of COVID-19 infection. While following up, if there was any suspicion for COVID-19 infection, a non-contrasted chest CT scan and RT-PCR test from a nasopharyngeal and oropharyngeal swab was completed.

For patients with postoperative COVID-19 infection, the percentage of lung involvement, re-hospitalization, ICU admission due to COVID-19, and its complications and mortality were recorded.

For statistical analysis, the application SPSS version 23 was used. Continuous data was reported as mean ± standard deviation while discrete data was reported as percentages and frequencies. Independent *t*-test was used to compare continuous data, whereas chi-square test and Fisher's exact test was used to compare discrete data.

## 3 Results

After excluding patients with pre-operative COVID-19 infections and those with the mentioned exclusion criteria, a total of 183 COVID-19 negative patients who underwent an operation were enrolled in this study. 98 participants (53.6%) were female, and 85 participants (46.4%) were male. Furthermore, the mean age within the sample was 47.06 ± 16.54 years and the mean body mass index (BMI) of the participants was 25.79 ± 4.29. 76 patients (41%) were overweight, 24 (13.3%) were obese, 75 (41%) were within the normal BMI range, and 8 (4.47%) were underweight. The mean pre-operative hospital admission was 1.74 ± 2.28 days. The indication and type of surgeries included as; Head & neck cancer 20 (11%), Upper GI cancer 10 (5.5%), Hepatobiliary cancer 20 (11%), Breast cancer 25 (13.5%), Colorectal cancer 8 (4%), Gynecologic cancer 4 (2%), Cholecystectomy 38 (21%), Appendectomy 16 (11.5%), Liver transplant 1 (0.5%), Laparotomy due to peritonitis or obstruction 16 (8%), Tissue repair after traumatic lacerations 11 (5.3%) and Coronary artery bypass graft 14 (6.7%).

Among the participants, 27 (17.5%) had diabetes mellitus, 40 (21.9%) had hypothyroidism, 32 (17.5%) had ischemic heart disease, 4 (2.2%) had renal disease, 56 (30.6%) had cancer, and 6 (3.3%) had respiratory disease. 80 patients (9.8%) underwent chemotherapy within three weeks pre-operation. 106 patients (57.9%) did not have any underlying conditions or comorbidities. Moreover, 132 patients underwent an elective operation, and 51 patients underwent a non-elective operation. During the pre-operation admission, it was noted that 1 patient had chronic myeloid leukemia (CML) and underwent an elective open splenectomy associated with a resultant prolonged hospital stay (44 days). Thus, this patient was excluded from the study (Table 1).

**Table 1:** Tabulation of post-operative COVID-19 status of surgical patients and their demographic information as well as comorbidities.

Variable	Total patients n = 183 (100%)	COVID-19 (−) patients n = 171 (93.4%)	COVID-19 (+) patients N = 12 (6.6%)	P-Value
**Diabetes Mellitus**	27 (14.8%)	26 (15.2%)	1 (8.3%)	1.000
**Hypertension**	40 (21.9%)	39 (22.8%)	1 (8.3%)	0.468
**Ischemic Heart Disease**	32 (17.5%)	30 (17.5%)	2 (16.7%)	1.000
**Renal Disorder**	4 (2.2%)	4 (2.3%)	0 (0%)	1.000
**Steroid Use**	7 (3.8%)	6 (3.5%)	1 (8.3%)	0.383
**Cancer**	56 (30.6%)	53 (31%)	3 (25%)	0.758
**Recent Chemotherapy**	18 (9.8%)	15 (8.8%)	3 (25%)	0.100
**Respiratory Disorder**	6 (3.3%)	5 (2.9%)	1 (8.3%)	0.338
**No Comorbidity**	106 (57.9%)	99 (57.9%)	7 (58.3%)	1.000
**Surgical priority**				
Elective	132 (72.1%)	120 (70.1%)	12 (100%)	0.022
Emergent	51 (27.9%)	51 (29.8%)	0 (0%)	
**Age (Mean ± SD, Years)**	47.7 ± 16.54	49.86 ± 16.05	49.17 ± 13.69	0.135
**Gender**				
Male	85 (46.4%)	78 (45.6%)	5 (41.6%)	0.289
Female	98 (53.6%)	93 (54.3%)	7 (58.3%)	
**Body Mass Index (Mean ± SD)**	25.70 ± 4.29	25.70 ± 4.49	26.39 ± 5.81	0.241
**Body Mass Index (Categorical)**				
Underweight	8 (4.4%)	8 (4.7%)	0 (0%)	0.876
Normal	75 (41%)	70 (40.9%)	5 (41.7%)	
Overweight	76 (41.5%)	71 (41.5%)	5 (41.7%)	
Obese	24 (13.1%)	22 (12.9%)	2 (16.7%)	
**Pre-operative hospitalization days (Mean ± SD)**	1.73 ± 2.24	1.74 ± 2.28	1.55 ± 1.29	0.434
**Operation time (Mean ± SD)**	155.6 ± 110.3	152.5 ± 109.5	200.7 ± 116.2	0.932

In the postoperative follow-up, 12 patients were positive for COVID-19 infection as identified by RT-PCR and non-contrasted chest CT scans. Regrettably, 2 individuals passed with one of these individuals dying as a direct result of COVID-19 infection. All the 12 cases of post-operative COVID-19 patients underwent elective surgeries and the mean age of patients in this group was 49.17 ± 13.69 years while those with no post-operative COVID-19 had a mean age of 46.99 ± 16.74 years, demonstrating no significant difference in age between the two groups. The mean operation time in the COVID-19 positive group was 200.75 ± 116.25 min, while this value was 152.5 ± 109.5 min in the COVID-19 negative group. Moreover, no significant difference was observed between the two groups in terms of BMI and sex distribution. The mean hospital stay duration before the operation was 1.55 ± 1.29 days in the COVID-19 positive group, while this value was 1.74 ± 2.28 days in the healthy group, once again demonstrating no significant difference between the two groups.

However, operation time was significantly higher in elective surgeries. (176.7 ± 115.3 min and 101.1 ± 72.4 min, P-value = 0.001). Interestingly, the distribution of underlying diseases, such as diabetes, hyperthyroidism, ischemic heart disease, renal disorders, cancer, respiratory disorders, and recent chemotherapy and steroid consumption, did not show a significant difference between the post-operative COVID-19 positive and negative groups.

## 4 Discussion

The COVID-19 pandemic significantly affected the efficacy of health care systems as well as the degree and type of care provided to patients all over the world [22]. Moreover, the fear of post-operative complications due to COVID-19 significantly limited and continues to limit both elective and urgent operations [9]. Many patients postponed their medical visits and admission due to concerns about COVID-19 infection [23]. These delays, whether because of patient or physician concerns or because of the devotion of supplies to COVID-19 management, could result in increased mortality and morbidity globally and significantly reduce patient quality of life due to delayed medical treatment [24,25].

Thus far, some studies report a high risk of COVID-19 infection and its related morbidity and mortality in hospital settings which suggests the need for careful observation of patients and the use of appropriate guidelines [26]. Thus far, literature suggests various directions for COVID-19 management in the surgical system including canceling all elective operations or observing tight protocols by operation room personnel [27]. Mihalj et al. suggest that PCR tests be used for the screening of all referral patients from other centers and all symptomatic patients before elective surgery in addition to PCR tests and chest CT scans for urgent cases [28]. Furthermore, De Simone et al. have provided a comprehensive approach for the management of COVID-19 infection in surgical patients and have suggested PCR tests and chest CT scans for all urgent cases and isolation of patient until screening test results are ready [29].

In our medical center, operations were performed by complete observance of pre-operative, intraoperative, and postoperative protocols. All patients were screened for COVID-19 before operations, and symptomatic patients were tested using RT-PCR. Visiting the patients was only allowed by medical staff to prevent viral spread. All patients were obligated to wear face masks except those requiring oxygen supplements or patients with dyspnea and all healthcare providers always wore face masks and observed infection protection protocols. In our center, no routine screening for COVID-19 was done for healthcare providers, and only symptomatic healthcare staff were evaluated for COVID-19. To add, patients with COVID-19 were admitted to separate wards and operated on in specifically allocated operating rooms. Finally, based on hospital protocol, it was ensured that both pre-operative and postoperative hospital stay were reduced as much as possible.

In a study by Axiotakis et al. study 9 cases out of 511 patients undergoing operation were infected with COVID-19. Prior to implementing pre-operative testing in their surgical center. Furthermore, they noted that the rate of COVID-19 infection has been reported to be related to Diabetes and ischemic heart disease [30].

In our study, 12 patients became symptomatic and infected with COVID-19, 2 of whom passed, 1 as a direct result of COVID-19. Thus, the observed mortality rate was less than that observed in similar studies (8.3%). In the Aminian et al. study during the onset of the pandemic, it was reported that three patients developed COVID-19 after the operation with 2 of these individuals passing [31]. In this study, no relationship was observed between underlying diseases and COVID-19 infection in the post-operative period; though, due to the risk of severity and mortality of COVID-19 in patients with comorbidity in the previous studies [11,32,33], it is essential to consider underlying diseases when planning an operation. Moreover, despite the results cited in previous studies, our study did not show a significant difference between genders in post-operative COVID-19 infection [33].

In our study, the incidence of post-operative COVID-19 infection only occurred in patients undergoing elective surgeries. This may be due to the simpler procedures, shorter hospitalization time, and the limited number of patients in non-elective surgeries and can be explained based on the significant difference observed between elective and non-elective operation duration. Although the present study did not show any relationship between the pre-operative length of stay and COVID-19 infection, reduced viral exposure due to observing preventive protocols and reducing the length of hospital stay where possible is key in reducing the risk of COVID-19 and further studies are required to clarify and amend guidelines [34].

One-third of the patients in the present study had cancer. Postponing elective but necessary surgeries for malignant diseases during the COVID-19 pandemic has resulted in increased mortality and morbidity due to metastatic and inoperable cases [34]. Considering the reduced risk of COVID-19 infection with the performed preventive measure and the risk of delaying operation in cancer patients, undergoing an operation while observing protocols is likely more beneficial for this group of patients [35,36].

The limitations of this study include the small sample size and the resulting low number of COVID-19 cases, hence, reducing the study's power and generalizability. Furthermore, it must be noted that non-inclusion of asymptomatic COVID-19 patients who presented with negative RT-PCR and no abnormal findings in the chest CT scans is also a limitation. Furthermore, future investigations comparing the postoperative complications between patients with COVID-19 and healthy individuals should also be conducted and will help shed light on the risks and benefits involved in conducting operations during the pandemic for policy makers.

Despite the mentioned limitations, the gathered results indicate a need for the re-evaluation of the risks of operation during the COVID-19 pandemic. If operations are performed while observing protective and preventative protocols, the risk of post-operative nosocomial COVID-19 are significantly reduced. Hence, the risks and the reduction in the quality of care provided and patient quality of life by the delay or cancellation of operations (most notably in cancer cases) may outweigh the risk of post-operative COVID-19 infections although a case-by-case analysis of the risks and comorbidities of each patient is likely required.

## Ethical approval

Ethical approval was requested and obtained from the “Tehran university of medical sciences” ethical committee.

## Source of funding

Tehran university of medical sciences.

## Author contribution

All authors contribute in this study equally.

## Consent

This study did not interfere with patients care but all patients was infirmed and consent was obtained.

## Registration of research studies

1. Name of the registry:
2. Unique Identifying number or registration ID:
3. Hyperlink to your specific registration (must be publicly accessible and will be checked):

## Guarantor

Mohammad Ashouri.

## Provenance and peer review

Not commissioned, externally peer reviewed.

## Declaration of competing interest

The authors have no conflict of interest.

## References

[1] Zhu N. (2020). A novel coronavirus from patients with pneumonia in China, 2019. N. Engl. J. Med..

[2] World Health Organization World Health Organization (2020). Coronavirus Disease 2019 (COVID-19): situation report e 66. https://www.who.int/emergencies/diseases/novel-coronavirus-2019/situation-reports.

[3] Wang D. (2020). Clinical characteristics of 138 hospitalized patients with 2019 novel coronavirus–infected pneumonia in wuhan, China. JAMA.

[4] Ghadir M.R. (2020). The COVID-19 outbreak in Iran; the first patient with a definite diagnosis. Arch. Iran. Med..

[5] Mercier G., Arquizan C., Roubille F. (2020). Understanding the effects of COVID-19 on health care and systems. Lancet Public Health.

[6] Dodangeh M., Dodangeh M. (2020). Iranian healthcare system against COVID-19. Germs.

[7] The Lancet R. (2021). Too long to wait: the impact of COVID-19 on elective surgery. The Lancet Rheumatol..

[8] Stöß C. (2020). The COVID-19 pandemic: impact on surgical departments of non-university hospitals. BMC Surg..

[9] Besnier E., Tuech J.-J., Schwarz L. (2020). We Asked the experts: covid-19 outbreak: is there Still a Place for scheduled surgery? “Reflection from pathophysiological data”. World J. Surg..

[10] Gangakhedkar G.R. (2020). Hazardous postoperative outcomes of unexpected COVID-19 infected patients: a call for global consideration of sampling all asymptomatic patients before surgical treatment. World J. Surg..

[11] Nepogodiev D. (2020). Mortality and pulmonary complications in patients undergoing surgery with perioperative SARS-CoV-2 infection: an international cohort study. Lancet.

[12] Flemming S. (2020). Surgery in times of COVID-19-recommendations for hospital and patient management. Langenbeck's Arch. Surg..

[13] American College of Surgeons (2020). https://www.facs.org/covid-19/clinical-guidance/nov2020-roadmap#viewport.

[14] American College of Surgeons (2020). https://www.facs.org/covid-19/clinical-guidance/elective-surgery.

[15] Finley C. (2020). Guidance for management of cancer surgery during the COVID-19 pandemic. Can. J. Surg..

[16] Moletta L. (2020). International guidelines and recommendations for surgery during Covid-19 pandemic: a Systematic Review. Int. J. Surg..

[17] Coccolini F. (2020). Surgery in COVID-19 patients: operational directives. World J. Emerg. Surg..

[18] Keramati M.R. (2020). Management of colon and rectal cancers during COVID-19 pandemic: a clinical guideline (TUMS-CRC-CoV19 Guideline). Med. J. Islam. Repub. Iran.

[19] Puylaert C.A.J. (2020). Yield of screening for COVID-19 in asymptomatic patients before elective or emergency surgery using chest CT and RT-PCR (SCOUT): multicenter study. Ann. Surg..

[20] Urban M.J. (2020). Implementation of preoperative screening protocols in otolaryngology during the COVID-19 pandemic. Otolaryngol. Head Neck Surg..

[21] Mathew G., Agha R., for the STROCSS Group (2021). Strocss 2021: strengthening the Reporting of cohort, cross-sectional and case-control studies in Surgery. Int. J. Surg..

[22] Roy C.M. (2021). Assessing the indirect effects of COVID-19 on healthcare delivery, utilization and health outcomes: a scoping review. Eur. J. Publ. Health.

[23] Nab M. (2021). Delayed emergency healthcare seeking behaviour by Dutch emergency department visitors during the first COVID-19 wave: a mixed methods retrospective observational study. BMC Emerg. Med..

[24] Spencer K. (2021). The impact of the COVID-19 pandemic on radiotherapy services in England, UK: a population-based study. Lancet Oncol..

[25] Tam C.F. (2020). Impact of coronavirus disease 2019 (COVID-19) outbreak on ST-segment-elevation myocardial infarction care in Hong Kong, China. Circ. Cardiovasc. Qual. Outcom..

[26] Nahshon C. (2020). Hazardous postoperative outcomes of unexpected COVID-19 infected patients: a call for global consideration of sampling all asymptomatic patients before surgical treatment. World J. Surg..

[27] Brindle M.E., Gawande A. (2020). Managing COVID-19 in surgical systems. Ann. Surg..

[28] Mihalj M. (2021).

[29] De Simone B. (2021). The management of surgical patients in the emergency setting during COVID-19 pandemic: the WSES position paper. World J. Emerg. Surg..

[30] Axiotakis L.G. (2021). Risk of acquiring perioperative COVID-19 during the initial pandemic peak: a retrospective cohort study. Ann. Surg..

[31] Aminian A. (2020). COVID-19 outbreak and surgical practice: unexpected fatality in perioperative period. Ann. Surg..

[32] Doglietto F. (2020). Factors associated with surgical mortality and complications among patients with and without coronavirus disease 2019 (COVID-19) in Italy. JAMA Surg..

[33] Nachon-Acosta A. (2021). Surgical outcomes during COVID-19 pandemic in a third level social security hospital in southeast Mexico. Arch. Med. Res..

[34] Du Q. (2021). Nosocomial infection of COVID-19: a new challenge for healthcare professionals (Review). Int. J. Mol. Med..

[35] Maringe C. (2020). The impact of the COVID-19 pandemic on cancer deaths due to delays in diagnosis in England, UK: a national, population-based, modelling study. Lancet Oncol..

[36] Riera, R., et al., Delays and disruptions in cancer health care due to COVID-19 pandemic: systematic review*.* JCO Global Oncol., 2021(7): p. 311-323. 10.1200/GO.20.00639 PMC8081532 33617304

